# TGF-β signaling induces SnoN to suppress BMP-induced hypertrophic maturation of chondrocytes

**DOI:** 10.1186/ar3642

**Published:** 2012-02-09

**Authors:** Shingo Maeda, Ichiro Kawamura, Yasuhiro Ishidou, Katsuyuki Imamura, Masahiro Yokouchi, Setsuro Komiya

**Affiliations:** 1 Department of Medical Joint Materials, Kagoshima University, Kagoshima, 890-8544, Japan; 2 Department of Orthopaedic Surgery, Kagoshima University, Kagoshima, 890-8544, Japan

## Background

Loss of TGF-β signaling in mice leads to promoted hypertrophic conversion of articular chondrocytes, which process is suggested to be linked to progression of osteoarthritis (OA). However, the molecular mechanisms by which TGF-β signaling inhibits chondrocyte maturation remain unclear. We screened for mediators downstream of TGF-β signaling to inhibit chondrocyte hypertrophy.

## Materials and methods

We induced choncrocyte differentiation of ATDC5 cells with BMP-2. A TGF-β type I receptor inhibitor compound SB431542 was applied to inhibit endogenous TGF-β signaling. Expression of differentiation markers was evaluated by real-time RT-PCR and immunoblot. The function of SnoN was studied by stable overexpression and siRNA-knockdown approaches. Organ culture system using mouse embryo metatarsal bone was employed to study the roles of TGF-β signaling and SnoN in chondrocyte maturation.

## Results

BMP-induced expression of *Col10a1 *gene, a specific marker for hypertrophic chondrocytes, was further up-regulated dramatically, upon treatment with SB431542. In metatarsal bone organ culture, zone of calcified matured chondrocytes was expanded upon SB431542 application. Expression of *Id1 *gene, the direct target of BMP Smads, was enhanced by SB431542, although the phosphorylation (activated status) of BMP-Smads-1/5/8 was not influenced by SB431542 application. Therefore, BMP signaling seemed to be blocked by TGF-β signaling at the level beneath the phosphorylation process of BMP-Smads. We evaluated expression profile of BMP signal-inhibitors, and found that *SnoN *was the only gene which expression was induced upon TGF-β treatment, while was inhibited by SB431542 application. Indeed, knockdown of SnoN resulted in enhanced hypertrophic maturation of ATDC5 cells, and overexpression of SnoN suppressed it. To evaluate *in vivo *contribution of SnoN in cartilage cell hypertrophy, we studied expression of SnoN protein by immunohistochemistry. In mouse growth plate, SnoN was present only in prehypertrophic chondrocytes, but excluded from hypertrophic zone. In human OA specimens, SnoN was positive around ectopic hypertrophic chondrocytes of moderate OA cartilages, whereas SnoN was not detected in severe-graded OA cartilages. These data support the idea that SnoN inhibits hypertrophic conversion of chondrocytes *in vivo*, as well as *in vitro*.

## Conclusions

Our results suggest that SnoN suppresses hypertrophic transition of chondrocytes, as a mediator of TGF-β signaling, to prevent the progression of OA.

